# Different Lengths of Diet Supplementation with 10% Flaxseed Alter the Hormonal Profile and the Follicular Fluid Fatty Acid Content of Fattening Gilts

**DOI:** 10.3390/life14020240

**Published:** 2024-02-08

**Authors:** Martina Lecová, Diana Babjáková, Drahomíra Sopková, Zuzana Andrejčáková, Zdenka Hertelyová, Vladimír Petrilla, Magdaléna Polláková, Radoslava Vlčková

**Affiliations:** 1 Department of Biology and Physiology, University of Veterinary Medicine and Pharmacy in Košice, Komenského 73, 041 81 Košice, Slovakia; martina.lecova@student.uvlf.sk (M.L.); diana.stefancova@student.uvlf.sk (D.B.); drahomira.sopkova@uvlf.sk (D.S.); zuzana.andrejcakova@uvlf.sk (Z.A.); vladimir.petrilla@uvlf.sk (V.P.); magdalena.pollakova@uvlf.sk (M.P.); 2 Department of Experimental Medicine, Faculty of Medicine, Pavol Jozef Šafárik University in Košice, Šrobárova 2, 041 80 Košice, Slovakia; zdenka.hertelyova@upjs.sk

**Keywords:** dietary flaxseed, fatty acid, follicular fluid, steroids, gonadotropins, IGF-I, porcine ovary

## Abstract

The effect of 10% dietary flaxseed fed for 3 and 6 weeks on serum hormone levels of fattening gilts, the fatty acid (FA) follicular fluid (FF) composition of small and large antral follicles, and the steroidogenesis and IGF-I secretion by isolated small antral follicles and their response to regulatory hormones (LH, FSH, IGF-I) was studied using immunoassay and gas chromatography analyses. Both supplemental periods increased levels of P4 and IGF-I in blood serum. A shorter period inhibited steroidogenesis (P4, T, E2) and IGF-I secretion by small antral follicles, which was associated with decreased levels of monounsaturated FAs (MUFA) and preferred n-6 polyunsaturated FA (PUFA) metabolism. A longer period stimulated hormone secretion at elevated levels of saturated FAs (SFA) at the expense of MUFAs and PUFAs preferring the n-3 PUFA metabolism. Out of ovarian regulators, only LH and IGF-I were able to alter the secretion of steroids and IGF-I by small follicles of fattening pigs fed a basal diet. The effect of flaxseed on the secretion of follicular hormones after both supplemental periods was altered by all regulatory hormones in a dose-dependent manner. The level of SFAs and PUFAs in FF of large follicles increased with the length of flaxseed feeding, suggesting the suppression of ovulation.

## 1. Introduction

Flaxseed is considered one of the best functional food products. It is rich in α-linolenic acid (ALA), as well as phytoestrogens, proteins, fiber, and other components beneficial to the animal body. Studies have shown many of its benefits including treating and preventing cardiovascular diseases, toxic effects of oil-related environmental pollutants, and having hepatoprotective, hypocholesterolemic, anti-inflammatory, anticancer, and laxative effects [1,2,3,4].

Numerous studies have focused on its effect on animal reproduction, particularly the female reproductive system, via lignans, ALA, and their products. Sirotkin [5] reviewed the effect of flaxseed on ovarian growth, follicle development, puberty, reproductive cycles, ovarian cell proliferation and apoptosis, oogenesis, embryogenesis, and hormonal regulators. The stimulatory and inhibitory actions of flaxseed have mostly been demonstrated in rodents. In rats, 5% flaxseed reduced ovarian weight, delayed puberty, and prolonged the diestrus. On the other hand, the 10% dose was proven to cause higher ovarian weight, lengthen the estrus cycle, and cause earlier puberty. Furthermore, the offspring of the mothers fed the 10% dose had similarly higher ovarian and uterine weights, earlier puberty onset and their estrus cycle was lengthened with persistent estrus [6]. Haran et al. [7] pointed out histologically normal, active ovarian tissue with well-nourished blood capillaries and all stages of follicle development after the addition of flaxseed lignans to rats’ diet. In the studies on mice, adding 10% flaxseed to their diet for 6 weeks [8] proved to result in larger ovaries and primary follicles and stimulated the release of progesterone (P4) and estradiol (E2) by the ovaries, while the diet supplemented for 2 weeks [9] showed opposite effects as well as altered proliferation and apoptosis of ovarian structures. Similar results were shown in sucklings and weanlings after a 10- and 24-day application of 10% flaxseed, respectively [10]. A flushing diet containing ALA increased the number of preovulatory follicles in goats [11]. In mares, flaxseed in vitro reduced granulosa cell viability and the release of P4 and PGF_2α_ [12]. 

The beneficial effect of flaxseed is related to its protective ability against toxic substances. As shown in the study by Vlčková et al. [9], dietary flaxseed (2-week application) was able to mitigate or invert the pro-proliferative effects of xylene on secondary ovarian follicles but to support this effect in tertiary follicles. Moreover, flaxseed reduced the stimulatory effect of xylene on the P4 and E2 levels in the blood and their release by the ovaries. The in vitro study by Sirotkin et al. [12] on equine granulosa cells confirmed that flaxseed mitigated the toluene action on cell proliferation, apoptosis, and the release of oxytocin and PGF_2α_. A similar protective effect of flaxseed against toluene was proven on human ovarian cells indicating that flaxseed could be a bio-stimulator of human reproduction and a protector against adverse effects of oil-industry environmental contaminants on female reproduction [13].

Flaxseed and its active molecules are considered potentially useful for improving farm animal reproductive efficiency, and treatment of polycystic ovarian syndrome and ovarian cancer. The action of flaxseed’s major components (lignans and polyunsaturated fatty acids; PUFAs) can be mediated by changes in general metabolism, metabolic and reproductive hormones, their binding proteins, receptors, and several intracellular signaling pathways regulating cell proliferation, apoptosis, angiogenesis, and malignant transformation [5]. The study by Vlčková et al. [9] also showed the influence of gonadotropins on the release of ovarian hormones as well as their ability to naturally protect the murine ovaries against the effects of either flaxseed or xylene. The main hormones supporting steroidogenesis in the ovaries are superior pituitary hormones and autoregulatory-acting growth factors such as insulin-like growth factor-I (IGF-I) [14]. Therefore, the first aim of this study was to test the effect of 10% flaxseed dietary application to gilts for 3 and 6 weeks of their fattening period, the release of P4, testosterone (T), E2, and IGF-I by ovarian fragments cultured with the addition of various doses of follicle-stimulating hormone (FSH), luteinizing hormone (LH), and IGF-I (to ascertain the effect of consumed flaxseed in response to the superior and local regulators added) and without (to ascertain the effect of consumed flaxseed).

All the metabolic fatty acid (FA) and lignan actions can be reflected in the composition of body fluids, including blood plasma [8] and follicular fluid [15] with the latter altering in functions of the respective cells. The in vitro and in vivo studies indicate that functional FAs are essential for the proper development of follicles, oocyte maturation, endometrial receptivity, placental development, embryo development, lactation performance, and influence steroidogenesis in granulosa cells/corpus luteum as well as lipid composition of follicular fluid [16]. Follicular fluid (FF) is a product of granulosa cells, theca cells, and the transfer of blood plasma constituents that can cross the blood follicular barrier [15]. As the oocyte takes up lipids mainly from FF, changes in the FA content of FF have a strong impact on oocyte maturation [17] and early embryo development [18]. Metabolic changes in serum are reflected in certain metabolites in FF, which can reversely affect the granulosa cells’ activity as well as oocyte quality [15]. Because of this, FF is a key and dynamic component that reflects the developmental status of the follicle and the activity of its cells, including the secretion of prostaglandins and steroid hormones [16]. Baddela et al. [19] observed that elevated levels of non-esterified fatty acids (NEFAs) in the blood caused a dramatic increase in their concentrations in FF and were associated with female subfertility. Fatty acids are shown to be vital throughout the reproductive cycle. Delayed estrus, poor oocyte quality, low fertilization rate, abortion, and many others can result from the shortage or excessive lipid reserves [16]. The modification of PUFA metabolism in mice after more than 2 weeks of flaxseed consumption was shown with the alteration in the ovarian steroidogenesis and steroid receptors on the uterine cells [10]. The studies were performed mainly on ruminants and rodents reporting plasma FA or FF lipid composition. Incomplete data, to our knowledge, is present in pigs. Therefore, this study has also focused on the assessment of the normal ratios of FAs in the FF of fattening gilt ovaries and the differences in the FA content in the FF of small and large growing follicles. Moreover, dietary flaxseed with a high content of ALA was used to ascertain which FAs were modified in the FF of those follicles after different (3- and 6-week) periods of diet supplementation.

## 2. Materials and Methods

### 2.1. Animals, Housing, and Diets

In the experiment, 18 gilts in total (20-week-old Landrace breed) were used in different fattening periods of 3 and 6 weeks to obtain reproduction data. Animals were housed at the Pig Fattening and Slaughter Station Inc. (Spišské Vlachy, Slovakia) in pens (2 animals/pen) fitted with nipple drinkers and free access to water. The average room temperature was 18 °C and relative humidity was 60%. 

Animals were divided into 3 groups according to the diet supplementation period. The first group was fed a commercial feed mixture (Table 1; composition declared by the producer) for fattening pigs (control group; C; n = 6) at a dose of 3 kg/day (Dom krmív, Spišské Vlachy, Slovakia). The diet of the experimental groups consisted of the commercial feed mixture and 10% flaxseed as a supplement fed to the animals for 3 (F3; n = 6) and 6 (F6; n = 6) weeks of fattening. The nutritional composition of diets is shown in Table 2. Flaxseed (Dom krmív) used was in the Libra variety with a high content of ALA (see fatty acid composition in Table 2). 

### 2.2. Blood Collection and Necropsy

At the end of the experiment (after 3 and 6 weeks of diet supplementation), blood was taken from v. cava cranialis and stood for 1 h, then processed by centrifugation at 200× *g* for 15 min to obtain serum (stored at −18 °C) for later immunoassays. The animals (n = 6 for each group) were slaughtered and their ovaries were collected for culture processing and collection of follicular fluid to analyze fatty acids.

### 2.3. Preparation and Processing of Ovarian Fragment Culture

The ovaries (n = 6 for each group) were defatted and transported to the tissue culture laboratory in cooling containers (4 °C) in phosphate-buffered saline (PBS) within 30 min of slaughtering. The ovaries were washed several times in PBS followed by a sterile physiological solution (0.9% NaCl) and dissected using a multi-blade knife. About 3 mm wedge ovarian tissue sections each containing one 2 to 3 mm follicle (1 fragment per well) were placed into sterile 24-well plates (Greiner CELLSTAR^®^ Sigma-Aldrich, St. Louis, MO, USA; 1 mL well) with sterile culture medium (DMEM/F12 1:1, Sigma; 1 mL per well) supplemented with 10% fetal bovine serum (FBS; Sigma) and 1% antibiotic–antimycotic (ATB-ATM) solution (Sigma), incubated at 37.5 °C in 5% CO_2_ humidified air for 24 h. The culture medium was replaced by a fresh medium mixture (DMEM + FBS + ATB-ATM) and tissue sections were incubated for another 24 h with or without the addition of FSH (follicle-stimulating hormone from porcine pituitary, 50 IU; Sigma), LH (porcine luteinizing hormone, 100 IU; ProSpec-Tany TechnoGene Ltd., Rehovot, Israel), and IGF-I (insulin-like growth factor-I human, 100 UG; Sigma). The substances were dissolved in a culture medium and added to the culture plate wells immediately before the experiment at 0, 0.01, 0.1, and 1 IU/mL (FSH and IGF-I) and at 0, 0.1, 1, and 10 IU/mL (LH). After incubation, media from the culture plates were aspirated using a pipette with a sterile tip and frozen at −20 °C to await immunoassays.

### 2.4. Immunoassays

Concentrations of progesterone (P4; ng/mL), testosterone (T; ng/mL), estradiol-17β (E2; pg/mL), and IGF-I (ng/mL) were determined in 25–100 µL of both blood serum and culture medium. For the analysis of P4 and E2 radioimmunoassay (RIA), T chemiluminescent microparticle immunoassay (CMIA), and IGF-I immunoradiometric assay (IRMA) were used following the manufacturer’s instructions. Samples were measured in duplicates. The detailed procedure is described in the previous study [21].

### 2.5. Follicular Fluid Collection and Gas Chromatography

Both ovaries of each animal from each experimental group were used for the follicular fluid collection. The follicular fluid from size 3 and 5 mm follicles (n = 6 samples for each follicle size and animal group) was aspirated using an insulin syringe with a needle of 0.3 mm diameter (BD Micro-Fine Plus, Becton, Dickinson and Company, Franklin Lakes, NJ, USA) and stored at −70 °C until analysis. The extraction of fatty acids (FA) from follicular fluid and gas chromatography were performed based on the method of Folch, et al. [22]. Extracted lipids were transesterified to FA methyl esters (FAME) with sodium methanolate. The profile of FAME was established using a gas chromatograph (Agilent 7890A, Agilent, Santa Clara, CA, USA) equipped with a flame ionization detector. The concentrations of FA were expressed in mol%.

### 2.6. Statistical Analyses

The data were analyzed using GraphPad Prism 5.0 for Windows (GraphPad Software, San Diego, CA, USA) and expressed as the mean and standard error of the mean (SEM). Each measured parameter was calculated per one animal (n = 6 for each group). Differences between the experimental groups from controls were compared using a one-way analysis of variance (ANOVA) with Dunnett’s multiple comparison test. Differences between the experimental periods were compared using an unpaired *t*-test. Differences were considered significant at least at *p* < 0.05 and identified with asterisks (differences between the experimental periods) and upper indexes (differences to controls).

## 3. Results

### 3.1. Serum Hormones

**Figure 1:**
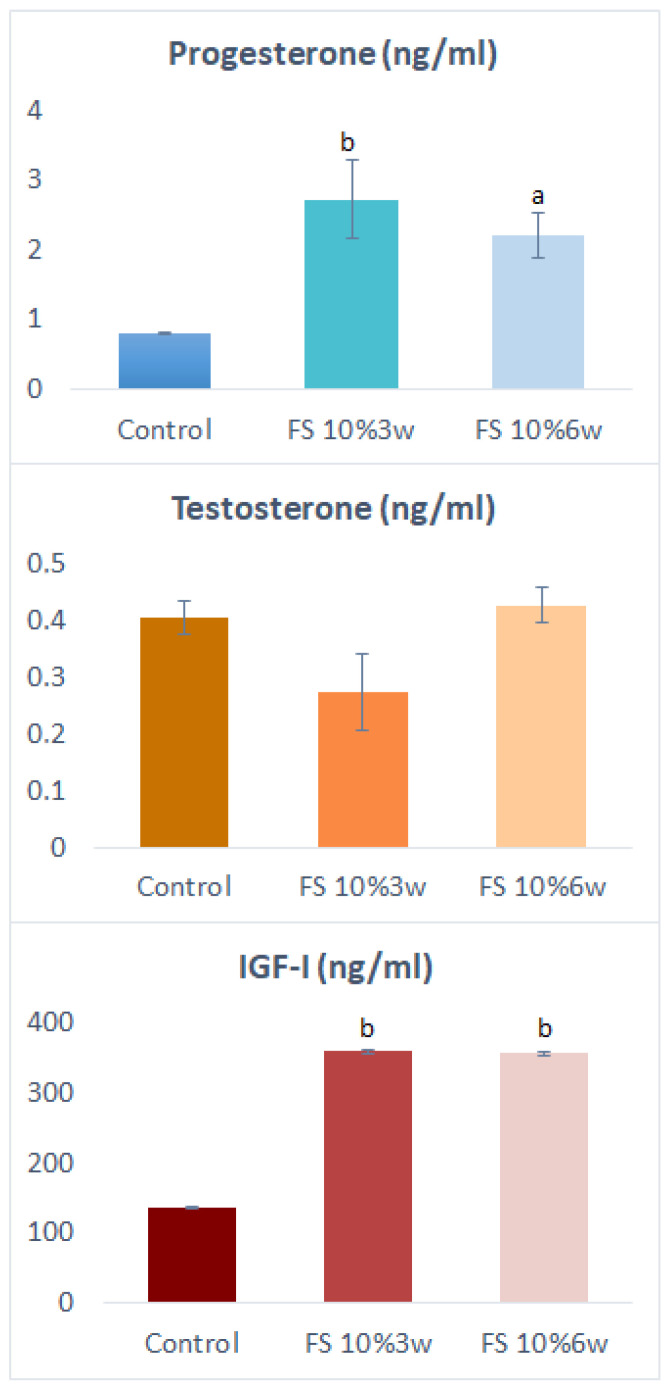
Hormone levels in blood serum of fattening pigs fed or not fed with 10% flaxseed. FS, flaxseed; IGF-I, insulin-like growth factor I; w, week. The values are mean ± SEM. ^a,b^—effect of the supplementation time length: significant (*p* < 0.05, *p* < 0.01) differences with control (no flaxseed administration).

The levels of progesterone (P4), testosterone (T), and IGF-I in the blood serum of fattening gilts fed with a diet supplemented with 10% flaxseed for different periods are shown in Figure 1. Estradiol-17β (E2) levels were lower than the detection limit (2 pg/mL) and therefore not reproducible. Flaxseed fed for both 3 and 6 weeks increased the levels of P4 (*p* ˂ 0.01 for 3 w; *p* ˂ 0.05 for 6 w) and IGF-I (*p* ˂ 0.01 for both 3 w and 6 w) but did not noticeably affect the level of T. There were no significant differences in the hormone levels between the experimental groups.

### 3.2. Culture Media Hormones

**Figure 2:**
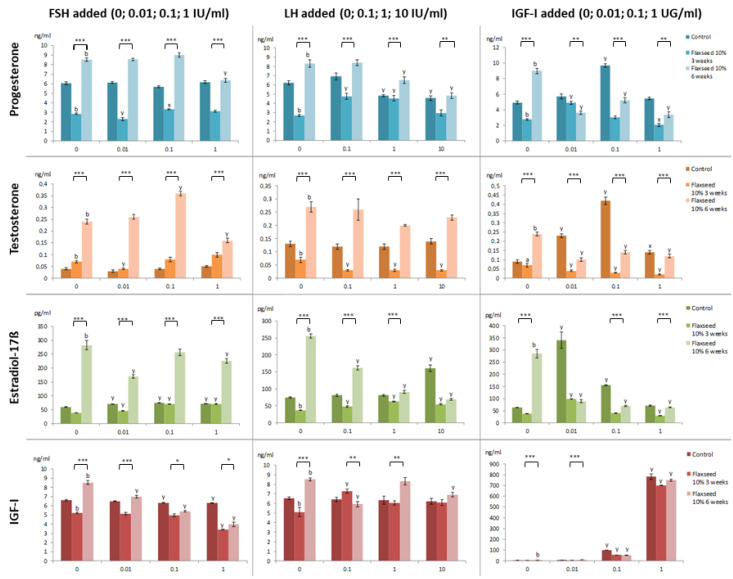
Hormone levels in culture media of small antral follicles of fattening pigs fed or not fed with 10% flaxseed without the addition of regulatory hormones (0 IU/mL or UG/mL) and after the addition of regulatory hormones (0.01, 0.1, and 1 IU/mL FSH; 0.1, 1, and 10 IU/mL LH; 0.01, 0.1, and 1 UG/mL IGF-I). FSH—follicle-stimulating hormone; LH—luteinizing hormone; IGF-I—insulin-like growth factor I; The values are mean ± SEM. ^a,b^—effect of the supplemental period length at no hormone addition (0 IU/mL or UG/mL): significant (*p* < 0.05, *p* < 0.01) differences with control (no flaxseed administration); ^x,y^—effect of regulatory hormones at different doses: significant (*p* < 0.05, *p* < 0.01) differences between the supplemental groups cultured with and without regulatory hormones; *, **, ***—effect of the supplementation time length: significant (*p* < 0.05, *p* < 0.01, *p* < 0.001) differences between different supplemental periods (6 weeks vs. 3 weeks).

The levels of P4, T, E2, and IGF-I in the culture media of ovarian fragments without (0 IU or UG; control) or with hormones (FSH, LH, IGF-I) added at different doses after feeding a diet supplemented with 10% flaxseed for different periods to fattening gilts are shown in Figure 2.

#### 3.2.1. Hormone Release without Any Regulatory Hormone Addition

The cultured ovary fragments decreased the secretion of P4 and IGF-I (both *p* ˂ 0.01) and increased the secretion of T (*p* ˂ 0.01) in the group of gilts fed with flaxseed-supplemented diet for 3 weeks (F3) compared to controls. Feeding the supplemented diet for 6 weeks (F6) resulted in the increased secretion of all ovarian hormones in comparison to the control (all *p* ˂ 0.01) and short supplemental period (all *p* ˂ 0.001).

#### 3.2.2. Hormone Release with the Addition of FSH

Follicle-stimulating hormone (FSH) added to the ovarian culture medium of the control group markedly increased the release of E2 at all doses (*p* ˂ 0.01) and decreased IGF-I at the doses 0.1 and 1 IU/mL (both *p* ˂ 0.01), while it did not alter the release of P4 and T.

In the F3 gilts’ ovarian fragments, FSH decreased the release of P4 and T at 0.01 IU/mL (both *p* ˂ 0.01) but slightly increased P4 at 0.1 IU/mL (*p* ˂ 0.05) and E2 at all doses (all *p* ˂ 0.01) in comparison to F3 fragments with no FSH addition. Other doses of FSH did not alter hormone secretion significantly. 

In the F6 group, FSH addition decreased the secretion of P4 and T (at 1 IU/mL; *p* ˂ 0.01), E2 (at 0.01 and 1 IU/mL; *p* ˂ 0.01), and IGF-I (at all doses; *p* ˂ 0.01), while increased the output of T at a dose of 0.1 IU/mL in comparison to F6 fragments with no FSH addition. Other doses of FSH did not alter hormone secretion significantly. 

When comparing the supplemental periods, the secretion of all hormones was significantly higher in F6 than in F3 ovarian fragments affected by the addition of FSH (at all doses *p* ˂ 0.001, except for IGF-I at 0.1 and 1 IU/mL; *p* ˂ 0.05). 

#### 3.2.3. Hormone Release with the Addition of LH

Luteinizing hormone (LH) addition to the ovarian culture medium of the controls (no supplemental diet) decreased the secretion of P4 at doses of 1 and 10 IU/mL (both *p* ˂ 0.01) and increased the secretion of E2 at 10 IU/mL dose (*p* ˂ 0.01) when compared to the control ovarian tissue without the addition of LH.

In the F3 gilts’ ovarian fragments, LH increased the output of P4 (at 0.1 and 1 IU/mL; *p* ˂ 0.01), E2 (at all doses; *p* ˂ 0.01), and IGF-I (at 0.1 IU/mL; *p* ˂ 0.01). Each added dose of LH decreased the output of T (all *p* ˂ 0.01) in comparison with the control F3 (no LH added). Higher doses of LH did not affect the secretion of P4 and IGF-I in this group.

In the F6 group, LH decreased the output of P4 (at 1 and 10 IU/mL; *p* ˂ 0.01), E2 (at all doses; *p* ˂ 0.01), and IGF-I (at 0.1 and 10 IU/mL; *p* ˂ 0.01) in comparison to the control F6 ovarian tissue with no LH addition. The lowest LH dose (0.1 IU/mL) and 1 IU/mL LH did not significantly affect the levels of P4 and IGF-I, respectively, secreted by the ovarian tissue. 

When comparing F6 to the F3 group, the addition of LH to the culture medium resulted in higher levels of all hormones (*p* ˂ 0.001; *p* ˂ 0.01 for P4 at 10 IU/mL and IGF-I at 0.1 and 1 IU/mL), except for IGF-I, which was lower than in F3 group at 0.1 IU/mL LH (*p* ˂ 0.01). The levels of E2 and IGF-I at the highest dose of LH (10 IU/mL LH) were comparable with that in group F3 (*p ˃* 0.05). 

#### 3.2.4. Hormone Release with the Addition of IGF-I

The insulin-like growth factor I (IGF-I) addition to the culture medium of the controls (no supplemental diet) increased the secretion of P4 (*p* ˂ 0.01 at 0.1 UG/mL), T and E2 (*p* ˂ 0.01 at 0.01 and 0.1 UG/mL), as well as IGF-I (*p* ˂ 0.01 at 0.1 and 1 UG/mL), as expected. 

In the F3 group, IGF-I added to the culture medium increased the release of P4 and E2 (*p* ˂ 0.01 at 0.01 UG/mL) as well as IGF-I (*p* ˂ 0.01 at 0.1 and 1 UG/mL), while it inhibited the secretion of P4 at the highest dose (1 UG/mL; *p* ˂ 0.01) and T at all doses (*p* ˂ 0.01) in comparison with the F3 group with no IGF-I addition.

In comparison to 0 UG/mL IGF-I, the cultivation of F6 ovarian fragments with IGF-I decreased the levels of P4, T, and E2 (all doses *p* ˂ 0.01), while it increased the levels of IGF-I secreted by F6 fragments at higher doses (0.1 and 1 UG/mL; *p* ˂ 0.01). The lowest dose of IGF-I (0.01 UG/mL) did not affect its secretion significantly. 

When comparing F6 to the F3 group, the level of P4 was lower at a dose of 0.01 UG/mL IGF-I (*p* ˂ 0.01), while it was higher at higher IGF-I doses (*p* ˂ 0.001 at 0.1 and *p* ˂ 0.01 at 1 UG/mL). The levels of T were clearly higher at all doses of additional IGF-I (all *p* ˂ 0.001). The levels of E2 were also higher in the F6 group (*p* ˂ 0.001 at 0.1 and 1 UG/mL IGF-I) but at 0.01 UG/mL IGF-I, the E2 level was comparable with that in the F3 group (*p ˃* 0.05). The levels of IGF-I did not significantly differ between the groups (*p ˃* 0.05).

### 3.3. Follicular Fluid Fatty Acids

The fatty acid (FA) profile of follicular fluid from 3 and 5 mm ovarian follicles obtained from fattening gilts on a 10% flaxseed supplemental diet for 3 and 6 weeks is shown in Table 3.

#### 3.3.1. Fatty Acids in 3 mm Follicles

Three weeks of diet supplementation with flaxseed compared to the control animals increased the levels of stearic (*p* ˂ 0.01), linoleic (LA; *p* ˂ 0.01), γ-linolenic (GLA; *p* ˂ 0.01), α-linolenic (ALA; *p* ˂ 0.01), dihomo-γ-linolenic (DGLA; *p* ˂ 0.01), and adrenic (*p* ˂ 0.01) FAs, and the total levels of polyunsaturated FAs (PUFA; *p* ˂ 0.01), n-3 (*p* ˂ 0.05) and n-6 (*p* ˂ 0.01) FAs, as well as the n-6: n-3 ratio (*p* ˂ 0.01). On the other hand, the levels of lauric (*p* ˂ 0.05), myristic, palmitic, palmitoleic, 7-hexadecenoic, vaccenic, oleic, gondoic, eicosadienoic, and cervonic FAs, and the total levels of unsaturated FAs (USFAs), monounsaturated FAs (MUFAs), n-7, and n-9 (all *p* ˂ 0.01) were decreased. The 3-week dietary flaxseed application did not significantly affect the levels of myristoleic, palmitelaidic, arachidic, mead, arachidonic (AA), timnodonic (EPA), osbond, and clupanodonic FA nor the total levels of saturated FAs (SFAs), trans-FAs, and the EPA: AA ratio.

Six weeks of diet supplementation with flaxseed compared to the controls resulted in an increase of lauric, myristic, myristoleic, palmitic, palmitelaidic, stearic, LA, GLA, and ALA (all *p* ˂ 0.01), and arachidic (*p* ˂ 0.05), mead (*p* ˂ 0.01), and clupanodonic (*p* ˂ 0.05) FA levels. The total levels of PUFAs, trans-FAs, n-3, and n-6 were also increased (all *p* ˂ 0.01). On the other hand, the levels of palmitoleic, 7-hexadecenoic, vaccenic, oleic, gondoic, eicosadienoic, DGLA, and AA (all *p* ˂ 0.01), cervonic (*p* ˂ 0.05) FA, and total levels of USFAs, MUFAs, n-7, n-9 (all *p* ˂ 0.01) were decreased compared to the controls. 

In comparison between the F6 and F3 group, the levels of lauric, myristic, myristoleic, palmitic, palmitelaidic, and stearic (all *p* ˂ 0.001), palmitoleic and mead FAs (both *p* ˂ 0.05), and total levels of SFAs, trans-FAs, (all *p* ˂ 0.001) were higher, while oleic, LA, gondoic, DGLA, AA, total levels of USFAs, MUFAs, PUFAs, n-6, the n-6: n-3 ratio, and n-9 FA (all *p* ˂ 0.001), and eicosadienoic (*p* ˂ 0.05) FA levels were lower. The levels of 7-hexadecenoic, vaccenic, GLA, ALA, arachidic, EPA, adrenic, osbond, clupanodonic, and cervonic FAs as well as the total levels of n-3, n-7 FA, and the EPA: AA ratio were not affected by the length of the supplemental period. 

#### 3.3.2. Fatty Acids in 5 mm Follicles

Three weeks of flaxseed supplementation compared to the control animals increased the levels of palmitic, stearic, LA, arachidic, mead, clupanodonic FAs, the total levels of SFAs, PUFAs, n-3, and n-6 FAs (all *p* ˂ 0.01), GLA, and the n-6: n-3 ratio (both *p* ˂ 0.05). Inversely, the levels of myristoleic, palmitoleic, 7-hexadecenoic, vaccenic, oleic, gondoic, and eicosadienoic FAs, and the total levels of USFAs, MUFAs, n-7, and n-9 (all *p* ˂ 0.01) were decreased. The short-term dietary flaxseed feeding did not markedly affect the levels of lauric, myristic, palmitelaidic, ALA, DGLA, AA, EPA, adrenic, osbond, and cervonic FAs nor the total levels of trans-FA and the EPA: AA ratio.

Six-week flaxseed supplementation resulted in the increase of lauric, myristic, myristoleic, palmitic, palmitelaidic, stearic, LA, arachidic, mead, EPA, and clupanodonic FA levels, the total levels of SFAs, PUFAs, trans-FA, n-3, n-6, the n-6: n-3 and EPA: AA ratios (all *p* ˂ 0.01), and GLA (*p* ˂ 0.05). Additionally, the levels of palmitoleic, 7-hexadecenoic, vaccenic, oleic, gondoic, eicosadienoic FAs, AA, and total levels of USFAs, MUFAs, n-7, and n-9 were decreased (all *p* ˂ 0.01) compared to the controls. 

Comparing F6 to the F3 group, the levels of lauric, myristoleic, palmitelaidic, stearic, LA, total levels of PUFAs, trans-FAs, the EPA: AA ratio (all *p* ˂ 0.001), as well as myristic FA and EPA (*p* ˂ 0.05) were higher in the F6 group. On the other hand, palmitoleic, 7-hexadecenoic, oleic, eicosadienoic FA, AA, MUFAs, and n-9 (all *p* ˂ 0.001) FA, osbond (*p* ˂ 0.05), and USFAs (*p* ˂ 0.01) levels were lower. The levels of ALA, DGLA, adrenic, and cervonic FAs were not affected by the length of the supplemental period.

## 4. Discussion

In this study, the effects of different time lengths of flaxseed supplementation on serum hormone levels, ovarian hormone secretion, their response to regulatory hormones, and the follicular fluid fatty acid composition in fattening gilts were investigated.

### 4.1. Can the Length of a Flaxseed Supplementing Diet Affect the Levels of Serum Hormones?

The addition of 10% flaxseed into the diet of gilts for 3 and 6 weeks increased the serum levels of P4 and IGF-I, but the level of T was comparable to the control (E2 levels were undetectable). There is a lack of information about the serum hormone composition after feeding flaxseed in pigs. The study in young gilts (sucklings and weanlings) [10] did not reveal an effect of dietary flaxseed on steroid hormone levels in the serum, but the level of IGF-I was increased, which was explained by its increased production in the liver during the growth phase of piglets. The findings in mice fed flaxseed for 2 weeks [9] were partially in line with those observed in the fattening gilts; P4 levels increased, but the effect on IGF-I levels was not proven. Many authors published their research in rats where dietary flaxseed increased P4 plasma levels [7,23,24,25] while decreasing T levels [23,24,25]. On the contrary, Riaz et al. [26] observed decreased levels of LH, FSH, P4, and E2, but the level of T conversely increased in female rats after treatment with flaxseed extract. Flaxseed fed to bovines brought more controversial results than in non-ruminant animals, as P4 serum levels both increased [27,28] and decreased [29] in cows fed with flaxseed. 

### 4.2. Can the Length of a Flaxseed Supplementation Period Modify the Hormone Secretion by the Ovaries?

In this study, flaxseed diet supplementation for 3 weeks inhibited the release of all hormones (P4, E2, T, and IGF-I) by ovarian fragments of fattening gilts, while a 6-week supplementation conversely stimulated the output of those hormones. These results are partially in line with previous studies in mice where flaxseed suppressed the release of ovarian P4 and E2, without a significant effect on IGF-I secretion after supplemental periods for a short time (2 weeks [8]) or a long time (6 weeks [9]) The 3-week supplementary period showed a similar inhibiting effect of flaxseed on P4 secretion as the direct effect of flaxseed extract on cultured porcine granulosa cells proven in the in vitro study by Sirotkin et al. [30] together with the stimulatory effect on the release of oxytocin and PGF_2α_. These findings suggest that such a hormonal status caused by flaxseed may threaten the lifespan of the corpus luteum and shorten the estrus cycle or potentially result in abortions.

### 4.3. Can Gonadotropins and IGF-I Modify the Release of Ovarian Hormones?

The development of growing antral follicles to preovulatory follicles is dependent on the gonadotropins’ actions. Follicle-stimulating hormone stimulates granulosa cells of such follicles and, together with IGF-I, induces cell proliferation and steroidogenesis supporting the secretion of FF and forming the follicular antrum [31]. Increasing secretion of E2 and its accumulation in the FF prepares the follicle to become dominant, to start producing P4, and to respond to the preovulatory release of LH from the anterior pituitary by subsequently triggering ovulation [32]. In the present study, FSH was only able to stimulate the secretion of E2 by small antral follicles. The higher-dose LH inhibited the release of P4 but promoted the release of E2 without altering T output, confirming its irreplaceable role in the follicle preparation for ovulation. On the other hand, IGF-I stimulated the output of all steroid hormones (P4, T, E2) and IGF-I itself, however, lower doses of IGF-I stimulated the secretion of P4 and E2 more than the higher ones. The in vitro experiment performed during this study has confirmed previous knowledge of the effects of gonadotropins on ovarian endocrine activity [33,34] and knowledge about the effect of IGF-I on the same activity. [35]. 

### 4.4. Can Supplementary Flaxseed Modify the Release of Ovarian Hormones in Response to Gonadotropins and IGF-I?

While a shorter (3-week) flaxseed supplemental period resulted in a decrease in all the ovarian steroids and IGF-I release, in response to gonadotropins and IGF-I added to the culture medium, their release was dose-dependent. In summary, the lowest dose of FSH suppressed the release of P4 and T, while the release of IGF-I was suppressed at the highest FSH dose. Conversely, FSH promoted the output of E2 at all doses but P4 only at higher doses. Luteinizing hormone supported the release of P4 and E2 at all doses, and IGF-I only at the lowest one, while it inhibited the secretion of T at all doses. Insulin-like growth factor-I promoted the secretion of P4 and E2 at the lowest and IGF-I only at higher doses, while it inhibited the secretion of P4 and E2 at the highest dose and T at all doses. Taken together, the addition of each regulatory hormone to the cultured ovarian fragments reversed the inhibitory effect of flaxseed on the release of P4 and E2 but supported it in the case of T. The highest dose of FSH supported the inhibitory effect of flaxseed on IGF-I secretion while LH and IGF-I reversed it. In the study where flaxseed was fed to mice for 2 weeks [9], the mixture of gonadotropins added to the ovarian tissue culture medium reversed the inhibitory effect of flaxseed on the P4 release and inhibited the stimulatory effect of flaxseed on the secretion of IGF-I but did not alter the secretion of E2. 

The 6-week flaxseed feeding resulted in the promotion of all ovarian steroids and IGF-I secretion, while the ovaries responded to hormone addition in a dose-dependent manner. Each regulatory hormone added to the ovarian culture mitigated the stimulatory effect of flaxseed on all the hormones’ secretion, while FSH was able to reverse this action on T at lower doses, and IGF-I, as assumed, promoted its stimulatory effect on IGF-I at higher doses. The response of ovaries to regulatory hormones was similar to that recorded after the short supplemental period; however, all the hormone levels were still higher. The results observed in the study performed on mice [8] were different regarding the FSH effect on the ovarian secretory activity after a 6-week supplemental period; FSH used in ovarian cultivation supported or inhibited the stimulatory flaxseed effect on the release of P4 or E2, respectively, with lower doses, while the effect on IGF-I secretion was not significant. The increased secretion of IGF-I by the porcine-growing antral follicles during long-term flaxseed feeding supports steroidogenesis at all levels of their metabolism, probably through the increased uptake of the substrate used for the synthesis of ovarian steroids and cholesterol. This probability can be supported by the studies focused on the effects of dietary flaxseed [2], in which the reduction of cholesterol levels in the blood was proven. 

### 4.5. Can the Length of a Flaxseed Supplementary Period Modify the Fatty Acid Composition of Follicular Fluid in Developing Follicles?

The physiological role of fatty acids is, among others, to ensure the fluidity of cell membranes as their components and to be precursors for steroid hormones and prostaglandins influencing the lipid composition of FF, follicular growth, and oocyte maturation [16].

In general, FF is rich in FAs, with non-esterified fatty acids (NEFAs) being the most prevalent. Their concentrations are affected by maternal physiological circumstances [36,37]. Palmitic (PA), stearic (STA), and oleic (OA) acids are considered to contribute up to 45% of the total NEFAs in FF [32] with PA being the most abundant in porcine oocytes [38]. In this study, the most pronounced FA levels in the FF of control fattening gilts were, in decreasing order, OA, PA, STA, LA, and AA, followed by ALA, other MUFAs (vaccenic, palmitoleic, and 7-hexadecenoic acids), and LCFAs [long-chain FAs; osbond acid, clupanodonic acid, DGLA (dihomo-γ-linolenic acid), EDA (eicosadienoic acid), DHA, and EPA] in both 3 and 5 mm follicles with OA, PA, and STA acids contributing up to 64%. This is a much higher percentage than was found in the previous study [32]. These authors revealed that elevated levels of NEFAs in circulation, in turn, enter the FF and alter their levels in developing ovarian follicles. Increased NEFA levels in FF then alter the functions of granulosa cells and oocytes. In bovine, murine, and human cumulus–oocyte complexes matured in vitro, the negative effect of high NEFA concentrations (PA, STA, and OA) was observed in increased glucose uptake and lactate production, accumulation of lipids, reduced mitochondrial membrane potential, and oxidative stress [39,40]. These can reduce oocyte developmental competence [41]. The cultivation of murine ovarian follicles in a high NEFA environment resulted in impaired ovarian steroidogenesis and oocyte competence for fertilization [37,41]. 

The data on levels of FAs obtained in the present study are not completely consistent with the study by Warzych et al. [42], who observed the primary FAs in porcine FF to be PA, OA, LA, STA, AA, ALA, DHA, and EPA with the levels of OA, ALA, EPA, and DHA lower and LA higher. Other studies also revealed that the major FAs in the FF of sows were PA, STA, and OA [36,39] and differed significantly with high and low reproductive performances [43]. Prepubertal pigs had the highest total FAs in FF, particularly PA and OA, while the FF of cyclic pigs contained more PUFAs LA and AA [44], but there is no data on the FA content in porcine antral follicles during their development. The present study revealed that the levels of FAs in the FF of porcine small and large antral follicles were almost identical. This is in line with the study performed in cyclic women [45], but the most pronounced FAs in FF were LA followed by PA, OA, STA, and AA, without a significant difference in their levels in both small and large follicles, although the AA: LA ratio was markedly higher in large follicles. It can be assumed that the development of antral follicles depends on the predominant type of FA specific to a certain animal species or breed and on the balance of FAs in the diet rather than on the type of FA specific to a certain follicle size. On the other hand, the dominance of follicles in ruminants [46,47] was specific to the increased concentration of ALA in FF. 

Although the data on FAs in the blood and FF among species fed flaxseed is quite well known from the literature (mares—[48]; livestock—reviewed in [16]), the data on the FA composition of FF in different-sized follicles is limited. In our study, 3 weeks of diet supplementation with flaxseed altered the FA profile in the FF of small antral follicles to the levels of LA, PA, STA, OA, AA, and ALA, in descending order. The 6-week supplemental period changed the order of FAs so that the level of PA exceeded the level of LA. The same scenario was noticed in 5 mm follicles after both 3 and 6 weeks of diet supplementation, but the levels of LA, ALA, DGLA, and AA were higher in the 3-week period than in the 6-week period. The study in mares fed flaxseed with salmon oil rich in n-3 PUFAs [48] revealed that the levels of LA, ALA, and DGLA in large follicles increased after 30 days of the diet supplementation, while 60-day supplementation resulted in the increased content of LA and AA and decreased those of ALA and DGLA to the control levels. Based on the present results, it can be assumed that adding flaxseed to the diet increases the content of SFAs (PA the most) and PUFAs (LA the most) while decreasing the content of MUFAs (OA the most) regardless of follicle size and the length of a supplemental period in fattening gilts. Diet supplementation with ALA protects oocyte developmental capacity under lipotoxic conditions [49] since ALA-rich diets could increase the levels of ALA in FF [50]. Although the ALA content in flax seed of pig supplemental diet was more than 50% of all PUFAs, its presence in FF was low, however, higher in small antral follicles than in large ones. 

The metabolism of n-6 and n-3 PUFAs depends on the activity of the delta-6-desaturase (Δ-6-D) enzyme, which catalyzes both types of PUFAs (LA and ALA) in the first step of their metabolic pathway in the formation of long-chain FAs (LCFA), such as AA and EPA and on the activity of the elongase enzyme. This is responsible for the prolongation of the carbon chain in the next step [51]. The amount of Δ-6-D is limited and its activity can be expressed as EPA: AA ratio [51]. In the present study, this ratio in FF of small and large ovarian follicles obtained from fattening gilts after 3 weeks as well as of small follicles after 6 weeks of flaxseed feeding reflected the higher uptake of Δ-6-D by the n-6 metabolism in favor of GLA synthesis. The ratio was elevated in the large follicles after a 6-week supplementary period reflecting the increased conversion of LA to GLA as well as ALA to EPA. Increased ingestion of LA (pro-inflammatory FA; [52]) from dietary flaxseed resulted in the higher synthesis of GLA (anti-inflammatory FA; [52]) in both small and large follicles. The difference was observed in further steps of the n-6 metabolic pathway, particularly in the elongation of GLA to DGLA as well as the desaturation of DGLA to AA, which was reflected in their levels in FF. Both DGLA and EPA are the precursor FAs for anti-inflammatory prostanoids (type 1 and 3 prostaglandins and leukotrienes, respectively) and AA is a precursor FA for pro-inflammatory prostanoids (type 2 prostaglandins and leukotrienes) [52]. It can be stated that shorter-term (3 weeks) flaxseed applications may promote the synthesis of anti-inflammatory prostanoids from DGLA and inhibit the synthesis of pro-inflammatory prostanoids from AA suppressing inflammation in small antral follicles. Moreover, about a 9-fold increase of adrenic acid, a metabolic product of AA, was noticed here, which is associated with the resolution of inflammation [53]. This is not true for large follicles, in which the levels of DGLA, AA, and EPA were not altered. On the other hand, long-term flaxseed applications may inhibit the synthesis of both anti- and pro-inflammatory prostanoids in small follicles. In large follicles, only pro-inflammatory prostanoids may be inhibited and increased EPA levels could potentially support the production of anti-inflammatory prostanoids. It is known that ovulation has attributes of inflammation including increased production of reactive oxygen species (ROS) and synthesis of prostaglandins [54], which may also be involved in follicular growth [45]. Therefore, insufficient production of pro-inflammatory substances (i.e., prostaglandin E2) may suppress the process of ovulation and ultimately cause fertility disorders. Our theory is supported by studies in women where after a 6-week receiving drink containing EPA and DHA, the content of these n-3 PUFAs in the FF increased together with clupanodonic acid (DPA-3) [55]. At the same time, LA and AA content was decreased. High levels of EPA, DPA-3, and DHA were observed to increase the chance of achieving pregnancy [56] and improve embryo quality [57,58]. Moreover, studies in ruminants [59,60] revealed that lower LA content in FF was associated with improved folliculogenesis, number of follicles, ovulation rate, IVF (in vitro fertilization) performance, and number of fetuses. This improvement was accompanied by a reduction of plasma cholesterol, estradiol, and insulin [61]. Further research is needed to clarify the mechanism by which dietary FAs alter folliculogenesis, ovarian steroidogenesis, ovulation process, and embryo quality.

Regarding ovarian steroidogenesis, LCFAs (e.g., AA and EPA) are considered precursors for the production of steroid hormones [16,19]. In the present study, together 24 different FAs were described in porcine FF, of which seven changed their distribution with the follicular size after a 3-week flaxseed supplemental period and only two after a 6-week period. From these observations, it is clear that feeding flaxseed in the diet for different lengths of time affects the presence of FAs in the FF of small antral follicles of fattening gilts and alters their endocrine activity. It was also noticed that 3 weeks of flaxseed feeding increased the content of some PUFAs (LA, ALA, GLA, DGLA, and adrenic acid) and STA, while decreasing the content of other SFAs (PA, myristic acid, lauric acid), MUFAs [OA, vaccenic acid, 7-hexadecenoic acid, palmitoleic acid (POA), gondoic acid], and PUFAs (eicosadienoid acid and DHA). These changes in FA composition of FF are related to the reduced ovarian steroid and IGF-I secretion. On the other hand, 6 weeks of flaxseed feeding changed the FA composition of FF in these follicles by increasing the content of SFAs (PA, STA, myristic acid, lauric acid), some MUFAs (POA, myristoleic acid, gondoic acid) and trans-FAs (palmitelaidic acid). The content of PUFAs (LA, AA, eicosadienoic acid, GLA) and the n-6: n-3 ratio decreased, which in turn stimulated steroidogenesis and IGF-I secretion. It has been established that SFAs are lipotoxic while USFAs are nontoxic and have favorable cellular functions [19,60,62,63]. Saturated fatty acids induce apoptosis of granulosa and cumulus ovarian cells in different species, which may further adversely affect the maturation and developmental competence of oocytes. Many types of USFAs affect proliferation and steroidogenesis processes and, especially when at elevated concentrations, could be detrimental to follicular cells [16,32]. The oleic acid-rich diet results in a decline in E2 and P4 production in porcine [64] and bovine [65] granulosa cells in vitro as well as in mice resulting in a shortening of estrus and a lower number of antral follicles and corpora lutea [64]. Furthermore, ALA and LA inhibited the expression of genes associated with granulosa cells’ steroidogenesis resulting in lower secretion of E2 and P4 [50]. High-dose AA, an n-6 LCFA derived in LA metabolism, suppressed the secretion of E2 but promoted the secretion of P4 by granulosa cells [66]. Conversely, DHA, an n-3 LCFA derived in ALA metabolism, promoted both the proliferation of bovine granulosa cells and the secretion of P4 and E2 [67]. In the case of fattening gilts, the flaxseed effect of both supplemental periods manifested in the increased levels of both LA and ALA in the FF of small antral follicles. However, after the 3 weeks, flaxseed inhibited secretion of steroids and IGF-I by these follicles, in which n-6 PUFA metabolism was stimulated more than n-3 PUFA metabolism resulting in a 9-fold increase in adrenic acid (n-6 LCFA) while decreasing DHA (n-3 PUFA). On the other hand, flaxseed fed for 6 weeks stimulated the secretion of follicular hormones at inhibited n-6 PUFA metabolism, decreasing AA levels while supporting n-3 PUFA metabolism with the accumulation of DPA (n-3 LCFA).

## 5. Conclusions

This study brings novel data on porcine ovary health influenced by 10% flaxseed consumption for various periods (3 and 6 weeks) including steroidogenesis in small antral follicles and fatty acid profiles in follicular fluid of various-sized antral follicles. It was observed that both lengths of supplemental periods resulted in increased levels of P4 and IGF-I in blood serum, but a shorter period inhibited while a longer period conversely stimulated steroidogenesis (P4, T, E2) and IGF-I secretion by small antral follicles. The study revealed that all the ovarian regulators (LH, FSH, and IGF-I) were able to alter the secretion of steroids and IGF-I by these follicles in pigs fed a basal diet or flaxseed-containing diet in a dose-dependent manner. Moreover, it was observed that FF contained almost identical levels of FAs among small and large antral follicles in fattening gilts fed on a basal diet with the most abundant being OA, PA, and STA followed by LA and AA. Dietary flaxseed supplementation changed the order of FAs in FF. A shorter period resulted in a 2-fold increase of LA making it the leading FA in small follicles and PA as a leader in large follicles. Palmitic acid was the most abundant in both types of follicles after the long-term diet supplementation with flaxseed. An increase in total SFAs after each flaxseed supplemental period in both small and large antral follicles was noticed where the rule was the longer the supplementation the higher the SFAs. The total MUFA levels were presented oppositely with a decreasing tendency the longer the supplementation lasted. The total PUFA levels were observed to be higher in small follicles after the shorter period and in large follicles after the longer diet fortification. 

It can be concluded that the 3-week period of flaxseed ingestion inhibits the secretion of steroids and IGF-I by small antral follicles, probably due to decreased levels of MUFAs and a preferred n-6 PUFA metabolism. On the other hand, 6 weeks of flaxseed feeding stimulates the secretion of the follicular hormones favoring SFAs at the expense of MUFAs and PUFAs preferring the n-3 PUFA metabolism. In both cases, such a fatty acid composition and metabolism could alter the production of anti-inflammatory prostaglandins from DGLA or decreased levels of AA on the side of n-6 metabolism as well as through the altered elongation of EPA to DHA on the side of n-3 metabolism. Since long-term diet supplementation with flaxseed stimulates steroidogenesis in small antral follicles, these could mature earlier than necessary. With a change in FA content of FF in a dominant stage, the ovulation process may be halted resulting in prolongation of the estrous cycle. This issue needs further experiments to clarify and understand the mechanisms of actions by which dietary flaxseed influences reproduction via fatty acids.

## Figures and Tables

**Table 1:** The ingredients (%, as fed) of the commercial complete feed mixture for fattening pigs.

Ingredient	%
Maize	13
Wheat	36.5
Barley	32
Soybean meal	8.5
Wheat bran	7
CaHPO_4_	0.78
CaCO_3_	1.29
NaCl	0.45
Mineral premix	0.48

**Table 2:** The analytical, nutrient, and fatty acid composition of the control and experimental diets (after Bartkovský et al. [20]).

Item	Basal Diet	Basal Diet + 10% Flaxseed
Analytical and nutrient composition *		
Crude protein (g/kg)	140.42	150.51
Crude fat (g/kg)	18.07	42.23
Crude fiber (g/kg)	35.69	68.29
Neutral detergent fiber (g/kg)	176.11	189.04
Acid detergent fiber (g/kg)	42.27	74.47
Ash (g/kg)	60.12	48.30
Starch (g/kg)	574.62	510.28
Ca (g/kg)	8.12	12.80
Mg (g/kg)	3.35	3.59
Na (g/kg)	1.45	1.52
K (g/kg)	5.58	6.29
P (g/kg)	4.80	5.84
Cu (mg/kg)	40.82	57.40
Zn (mg/kg)	131.83	103.45
Mn (mg/kg)	150.57	125.80
ME (MJ/kg)	13.26	13.36
**Fatty acid composition (%)**		
C14:0, Myristic acid	0.101	0.050
C16:00, Palmitic acid	14.281	4.496
C18:00, Stearic acid	1.899	2.547
C18:2n-6, Linoleic acid	55.730	8.547
C18:3n-6, Gamma-linolenic acid	0.090	0.018
C18:3n-3, Alpha-linoleic acid	7.109	72.546
C20:4n-6, Arachidonic acid	0.691	0.052
C20:5n-3, Eicosapentaenoic acid	0.125	0.0003
C22:5n-6, Docosapentanoic acid	0.245	0.022
C22:5n-3, Docosapentaenoic acid	0.043	0.140
C22:6n-3, Docosahexaenoic acid	0.073	0.055
Σ n-3	7.349	72.741
Σ n-6	57.227	8.720
n-6/n-3	7.787	0.120
EPA/AA	0.181	0.005

*—calculated to 100% of dry matter; ME—metabolizable energy; EPA—eicosapentaenoic acid; AA—arachidonic acid.

**Table 3:** Effect of dietary flaxseed on the levels of selected fatty acids in the follicular fluid of ovarian follicles (3 mm and 5 mm) of fattening pigs.

Fatty Acids (mol%)	Fatty Acid Trivial Name	None (Control) 3 mm F	Flaxseed 3 w/3 mm F	Flaxseed 6 w/3 mm F	None (Control) 5 mm F	Flaxseed 3 w/5 mm F	Flaxseed 6 w/5 mm F
C12:0	Lauric	0.09 ± 0.01	0.02 ± 0.00 ^a^	0.34 ± 0.03 ^bz^	0.09 ± 0.01	0.09 ± 0.01	0.44 ± 0.03 ^bz^
C14:0	Myristic	0.60 ± 0.03	0.42 ± 0.02 ^b^	0.94 ± 0.02 ^bz^	0.60 ± 0.03	0.70 ± 0.04	0.82 ± 0.04 ^bx^
C14:1n5	Myristoleic	0.06 ± 0.00	0.06 ± 0.00	0.10 ± 0.01 ^bz^	0.06 ± 0.00	0.03 ± 0.00 ^b^	0.11 ± 0.01 ^bz^
C16:0	Palmitic	23.47 ± 0.17	22.61 ± 0.16 ^b^	28.20 ± 0.17 ^bz^	23.48 ± 0.24	27.05 ± 0.21 ^b^	26.43 ± 0.24 ^b^
C16:1n7t	Palmitelaidic	0.11 ± 0.02	0.08 ± 0.00	0.28 ± 0.01 ^bz^	0.11 ± 0.00	0.11 ± 0.00	0.19 ± 0.01 ^bz^
C16:1n7	Palmitoleic	1.44 ± 0.02	0.65 ± 0.02 ^b^	0.73 ± 0.02 ^bx^	1.44 ± 0.02	0.71 ± 0.00 ^b^	0.98 ± 0.05 ^bz^
C16:1n-9	7-hexadecenoic	1.25 ± 0.00	0.81 ± 0.02 ^b^	0.72 ± 0.05 ^b^	1.25 ± 0.020	0.86 ± 0.02 ^b^	0.58 ± 0.02 ^bz^
C18:0	Stearic	14.72 ± 0.31	16.77 ± 0.26 ^b^	18.21 ± 0.17 ^by^	14.72 ± 0.20	15.77 ± 0.19 ^b^	16.85 ± 0.25 ^by^
C18:1n7	Vaccenic	2.91 ± 0.26	1.95 ± 0.23 ^b^	1.29 ± 0.12 ^b^	2.91 ± 0.22	1.46 ± 0.26 ^b^	1.11 ± 0.13 ^b^
C18:1n9	Oleic	25.84 ± 0.38	14.09 ± 0.24 ^b^	11.18 ± 0.19 ^bz^	25.84 ± 0.26	13.73 ± 0.30 ^b^	10.75 ± 0.24 ^bz^
C18:2n6	Linoleic	11.99 ± 0.28	24.15 ± 0.24 ^b^	21.95 ± 0.20 ^bz^	11.99 ± 0.38	20.43 ± 0.23 ^b^	24.91 ± 0.22 ^bz^
C18:3n6	γ-linoleic	0.03 ± 0.01	0.13 ± 0.02 ^b^	0.11 ± 0.02 ^b^	0.03 ± 0.01	0.08 ± 0.01 ^a^	0.08 ± 0.01 ^a^
C18:3n3	α-linolenic	1.19 ± 0.12	2.04 ± 0.01 ^b^	2.36 ± 0.01 ^b^	1.19 ± 0.03	1.53 ± 0.16	1.32 ± 0.10
C20:0	Arachidic	0.04 ± 0.01	0.06 ± 0.01	0.09 ± 0.01 ^a^	0.04 ± 0.01	0.11 ± 0.00 ^b^	0.09 ± 0.01 ^b^
C20:1n9	Gondoic	0.11 ± 0.01	0.07 ± 0.01 ^b^	0.01 ± 0.00 ^bz^	0.11 ± 0.00	0.03 ± 0.01 ^b^	0.01 ± 0.00 ^b^
C20:2n6	Eicosadienoic	0.45 ± 0.06	0.27 ± 0.02 ^b^	0.16 ± 0.00 ^bx^	0.45 ± 0.01	0.25 ± 0.01 ^b^	0.15 ± 0.01 ^bz^
C20:3n6	Dihomo-γ-linoleic	0.50 ± 0.01	0.61 ± 0.01 ^b^	0.41 ± 0.01 ^bz^	0.50 ± 0.10	0.58 ± 0.03	0.44 ± 0.03
C20:3n9	Mead	0.26 ± 0.01	0.31 ± 0.03	0.39 ± 0.02 ^bx^	0.26 ± 0.02	0.36 ± 0.01 ^b^	0.38 ± 0.01 ^b^
C20:4n6	Arachidonic (AA)	11.20 ± 0.03	11.02 ± 0.03	8.87 ± 0.15 ^bz^	11.20 ± 0.27	11.67 ± 0.20	9.81 ± 0.24 ^bz^
C20:5n3	Timnodonic (EPA)	0.27 ± 0.08	0.29 ± 0.09	0.23 ± 0.07	0.27 ± 0.10	0.31 ± 0.10	0.77 ± 0.10 ^bx^
C22:4n6	Adrenic	0.06 ± 0.02	0.54 ± 0.12 ^b^	0.30 ± 0.07	0.06 ± 0.01	0.33 ± 0.10	0.26 ± 0.09
C22:5n6	Osbond	1.26 ± 0.07	1.14 ± 0.05	1.09 ± 0.01	1.26 ± 0.09	1.31 ± 0.10	1.00 ± 0.00 ^x^
C22:5n3	Clupanodonic	1.20 ± 0.04	1.42 ± 0.11	1.57 ± 0.13 ^a^	1.20 ± 0.06	1.79 ± 0.10 ^b^	1.73 ± 0.15 ^b^
C22:6n3	Cervonic	0.95 ± 0.13	0.45 ± 0.09 ^b^	0.55 ± 0.08 ^a^	0.95 ± 0.08	0.70 ± 0.08	0.73 ± 0.09
ΣSFA		38.86 ± 0.34	39.55 ± 0.49	47.50 ± 0.29 ^bz^	38.81 ± 0.36	43.66 ± 0.18 ^b^	44.58 ± 0.18 ^b^
ΣUSFA		61.05 ± 0.06	60.04 ± 0.07 ^b^	52.20 ± 0.06 ^bz^	61.05 ± 0.05	56.24 ± 0.07 ^b^	55.52 ± 0.23 ^by^
ΣMUFA		32.03 ± 0.26	18.00 ± 0.26 ^b^	14.76 ± 0.12 ^bz^	31.94 ± 0.06	17.09 ± 0.13 ^b^	14.08 ± 0.14 ^bz^
ΣPUFA		29.95 ± 0.13	42.72 ± 0.12 ^b^	37.94 ± 0.13 ^bz^	29.82 ± 0.20	39.59 ± 0.14 ^b^	41.69 ± 0.18 ^bz^
ΣTRANS		0.11 ± 0.01	0.06 ± 0.01	0.26 ± 0.02 ^bz^	0.11 ± 0.01	0.11 ± 0.01	0.19 ± 0.01 ^bz^
Σn-3		3.60 ± 0.11	4.20 ± 0.18 ^a^	4.71 ± 0.17 ^b^	3.60 ± 0.14	4.33 ± 0.13 ^b^	4.55 ± 0.17 ^b^
Σn-6		25.75 ± 0.64	38.17 ± 0.67 ^b^	33.23 ± 0.50 ^bz^	25.75 ± 0.67	35.01 ± 0.35 ^b^	37.03 ± 0.62 ^b^
n-6: n-3		7.17 ± 0.16	9.14 ± 0.23 ^b^	7.11 ± 0.32 ^z^	7.18 ± 0.12	8.09 ± 0.27 ^a^	8.17 ± 0.22 ^b^
EPA: AA		0.02 ± 0.01	0.03 ± 0.01	0.03 ± 0.01	0.02 ± 0.01	0.03 ± 0.01	0.08 ± 0.01 ^by^
Σn-7		4.46 ± 0.23	2.67 ± 0.10 ^b^	2.30 ± 0.12 ^b^	4.46 ± 0.11	2.27 ± 0.06 ^b^	2.28 ± 0.12 ^b^
Σn-9		27.20 ± 0.54	14.97 ± 0.51 ^b^	11.91 ± 0.17 ^bz^	27.20 ± 0.34	14.62 ± 0.28 ^b^	11.35 ± 0.19 ^bz^

Values are mean ± SEM. F, follicle; SFA, saturated fatty acids; USFA, unsaturated fatty acids; MUFA, monounsaturated fatty acids; PUFA, polyunsaturated fatty acids; t, trans-fatty acids. Values are means ± SEM; ^a,b^ Mean values within rows differ significantly from corresponding controls, ^x,y,z^ Mean values within rows differ significantly between supplemental period, ^a,x^ *p* < 0.05; ^b,y^ *p* < 0.01; ^z^ *p* < 0.001.

## Data Availability

All data related to this study are presented in the main text.
